# Low Serum Alpha-Antitrypsin Associated with Anti-PR-3 ANCA in Autistic Children with GI Disease

**DOI:** 10.4137/gei.s2918

**Published:** 2009-06-04

**Authors:** AJ Russo, A Krigsman, B Jepson, Andrew Wakefield

**Affiliations:** 1 Research Director Health Research Institute/Pfeiffer Treatment Center, 4575 Weaver Parkway Warrenville, Illinois 60555.; 2 Thoughtful House Center for Children, 3001 Bee Caves Road, Austin, Texas, 78746.

**Keywords:** autism, PR3, alpha-1 antitrypsin, GI disease

## Abstract

**Aim:**

To assess the possible relationship between serum alpha-1 antitrypsin (AAT) levels and anti-neutrophil cytoplasmic antibodies (ANCA) in autistic children with severe GI disease and to test the hypothesis that there is an association between low serum AAT levels, the presence of ANCA and inflammatory GI disease seen in some autistic children.

**Subjects and Methods:**

Serum from 40 autistic children with chronic digestive disease (many with ileo-colonic lymphoid nodular hyperplasia (LNH) and inflammation of the colorectum, small bowel and/or stomach), and 41 controls (21 age matched autistic children with no GI disease and 20 age matched children without autism or GI disease) were tested using ELISAs designed to quantitate ANCA (anti-PR3), AAT and PR3 levels.

**Results:**

We found that a significant number of autistic children with chronic digestive disease had anti-PR3 ANCA, high serum PR3 and high severity of disease when compared to controls.

This same group of autistic children had low serum levels of AAT compared to controls, which also correlated with the presence of anti-PR3 ANCA, high serum PR3, as well as the severity of intestinal disease, particularly LNH and severe erythema.

**Discussion:**

These results suggest a relationship between low AAT levels, ANCA and severity of GI disease seen in a subpopulation of ASD individuals. We suggest that low AAT levels may result in high levels of PR3, which may, in turn be associated with the presence of ANCA.

## Introduction

Alpha-1-antitrypsin (AAT), a 394 amino acid, 52 kDa glycoprotein synthesized by the liver, is the most abundant circulating serine protease inhibitor with normal serum concentration of 85–250 mg/dL.^1^–^3^ AAT deficiency, caused by mutations in the AAT gene coding^4^–^6^ on the long arm of human chromosome 14 (locus 14q32.1),^7^ is a genetic condition that increases the risk of developing a variety of diseases including pulmonary emphysema, cirrhosis of the liver and gut disease. It is a naturally occurring inhibitor of proteinase 3 (PR3), one of the target antigens of anti-neutrophil cytoplasmic antibodies (cANCA).^8^,^9^ An increased incidence of alpha 1-AT phenotypes associated with dysfunctional alpha 1-AT or low serum levels has been reported in patients with anti-PR3 antibodies.^10^,^11^

cANCA were originally detected in serum from patients with Wegener’s granulomatosis (WG),^8^ a disease characterized by necrotizing granulomatous inflammation of the upper and lower airways in conjunction with systemic vasculitis and necrotizing crescentic glomerulonephritis.^12^ Following the detection of ANCA in systemic vasculitis, it became clear that ANCA also occurred in other idiopathic inflammatory disorders,^13^ including inflammatory bowel diseases or IBD (which include ulcerative colitis (UC) and Crohn’s disease (CD)),^14^,^15^ in autoimmune-mediated liver diseases,^16^–^18^ in rheumatoid arthritis (RA),^19^,^20^ and in systemic lupus eythematosus (SLE).^21^,^22^

Autistic Spectrum Disorder (ASD) is a neuro developmental syndrome with onset prior to age 36 months. Diagnostic criteria consists of impairments in sociality and communication plus repetitive and stereotypic behaviors.^23^ Traits strongly associated with autism include movement disorders and sensory dysfunctions.^24^ Although autism may be apparent soon after birth, many autistic children experience at least several months, up to a year or more in some cases, of normal development—followed by regression, defined as loss of function or failure to progress.^24^–^26^ Children with autistic spectrum disorders (ASD) frequently have accompanying gastrointestinal (GI) symptoms.^27^–^29^

We previously reported that, when compared to controls, a significant number of autistic family members have lower than normal serum levels of AAT.^36^ We have also reported that a significant number of autistic children with GI disease have ANCA (both anti-PR3 and anti-MPO (myeloperoxidase)), and that there is a relationship between individuals with ANCA and severity of intestinal disease.^37^ In this study, we report preliminary data suggesting that a significant number of autistic children with chronic digestive disease have low serum AAT, anti-PR3 ANCA and high serum PR3, which correlate with high severity of GI disease. These data, although not providing a causative relationship between these biomarkers and GI disease found in some autistic children, suggest that low AAT levels may result in high levels of PR3, which may, in turn be associated with the formation of ANCA.

## Materials and Methods

### ELISA to measure serum anti-PR3 antibodies and anti-MPO antibodies (IMMCO diagnostics, Buffalo, NY)

All reagents and specimens were equilibrated to room temperature before the assay was performed. A 1:51 dilution of the patient samples were prepared by mixing 10 μl of the patient sera with 0.5 ml of Serum Diluent. One hundred microliters of calibrators (20–200 Eu/ml antibodies), positive and Negative control serums, serum diluent alone, and diluted patient samples were added to the appropriate microwells of a microculture plate (each well contained approximately 150 ng of purified PR3 or MPO). Wells were incubate for 30 minutes (±5 min) at room temperature, then washed 4 × with wash buffer. One hundred microliters of pre-diluter anti-human IgG conjugated with alkaline phosphotase was added to all microwells, incubated for 30 minutes (±5 min) at room temperature, then wash 4 × with wash buffer. One hundred microliters of enzyme substrate was added to each microwell. After approximately 30 minutes at room temperature, the wells were read at 405 nm with an ELISA reader.

### ELISA to measure plasma PR3 levels

A microtitre plate was coated overnight with affinity-purified rabbit anti-PR3 diluted to 1/300 in sample buffer. Plasma samples diluted to 1/20 and 1/40 in sample buffer were added and the plates were incubated for 2 h. After washing, bound PR3 was detected by incubation for 2 h with a monoclonal PR3 antibody (Sigma). Washing was followed by the addition of the alkaline phosphatase conjugated goat anti-rabbit IgG (Biorad), diluted to 1/1000 in sample buffer and then incubated for 1 h at 40 C. After washing the wells 5X, alkaline phosphatase substrate was added and color development was mesasured using an ELISA reader.

### ELISA to measure serum alpha-1 antitrypsin (ALPCO)

Prior to use in the assay all reagents and samples were allowed to come to room temperature (18–26 C) and then mixed well. Each well was washed 5 times by dispensing 250 μl of diluted wash buffer into each well then removing residual buffer by tapping the plate on absorbent paper toweling. 100 μl of standards, samples and controls were dispensed into respective wells and the plate was incubated overnight at 4 C. The plate was washed 5 times as described above. 100 μl of anti-AAT conjugate was added to each well and the plate was incubated for 1 hour at room temperature. The plate was washed 5, 100 μl of substrate was added to each well and the plate was incubated at room temperature for 20 minutes. Absorption was determined with an ELISA reader at 450 nm.

### Subjects and scoring of severity of GI disease

Serum from autistic individuals with GI disease were obtained from the Thoughtful House, Austin, Texas. All 40 children with ASD (median age 6 years; range 2–16; 34 male) with gastrointestinal symptoms, who participated in this study, were investigated by ileo-colonoscopy. Macroscopic and histological features of the upper and lower GI tract were scored. A point system was developed to assess the *severity of GI disease* (particularly inflammation). Patients were scored according to mild (1 point), moderate (2 points) and marked (3 points) disease in each area (upper and lower GI) and for scope (macroscopic) and histology of each area. Therefore the maximum score for GI disease would be 12 (3 points each for upper scope, upper histology, lower scope and lower histology). A point system was also developed for severity of lymphoid nodular hyperplasia (LNH). Patients were scored according to mild (1 point), moderate (2 points) and marked (3 points) LNH in each area (upper and lower GI) for a maximum of 6 points. And finally, a point system was also developed for severity of erythema. Patients were scored according to mild (1 point), moderate (2 points) and marked (3 points) erythema in each area (upper and lower GI) for a maximum of 6 points.

### Controls

Two control groups (total n =41) were studied, including 21 age (mean 68 months), gender (80% male) and diagnosis (61% regressive onset) matched autistic children with no GI disease and 20 age (mean 71 months) and gender (75% male) matched children without autism or GI disease. Serum and medical history were obtained from the Autism Genetic Resource Exchange—AGRE*.

### Serums

Experimental and control serums were frozen at −70 C immediately after collection and separation.

#### Statistics

Inferential statistics were derived from t-test and odds ratios with 95% confidence intervals.

## Results

**Figure 1:**
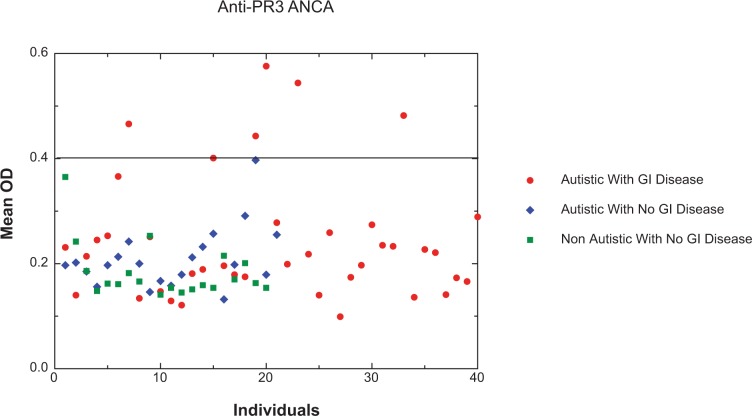
Scatter diagram showing ANCA in Autistic children with severe GI disease compared to controls. Forty autistic children with severe GI disease (A-GI) and 41 controls (including 21 age (mean 68 months), gender (80% male) and diagnosis (61% regressive onset) matched autistic children with no GI disease and 20 age (mean 71 months) and gender (75% male) matched children without autism or GI disease) were tested for anti-PR3 IgG. Six A-GI had anti-PR3 IgG (greater than 20 Eu/ml, above —), whereas only one of the controls had borderline anti-PR3 IgG. The data points represent the mean of at least 2 separate assays. The standard deviation (not shown) of each mean was less than +/− 0.01 OD.

Using an ELISA described above, 6 of 40 autistic children with chronic digestive disease had anti-PR3 antibodies compared to only one of the 41 controls (p < 0.01) (Fig. 1). Five of six individuals with anti-PR3 IgG also had high levels of PR3 (p < 0.05) (Table 1). Figure 4 shows the results of a typical assay measuring serum ANCA in autistic children with GI disease.

There was a significant relationship between individuals with ANCA and severity of disease, particularly the presence and severity of LNH and abnormal vascularization. Fifteen of the 40 autistic children with GI disease were categorized as having severe disease (score equal or greater than 7 on total GI severity score described above), 6 of 40 had severe LNH (score equal or greater than 4), and 5 of 40 had severe erythema (Table 1). Four (of 6) of the individuals with anti-PR3 IgG had severe Total GI disease (p < 0.01).

(Table 1). Three (of 6) of the children with severe LNH also had ant-PR3 IgG (p < 0.05), and 3 (of 5) children with severe erythema had anti-PR3 IgG (p < 0.05) (Table 1).

**Figure 2:**
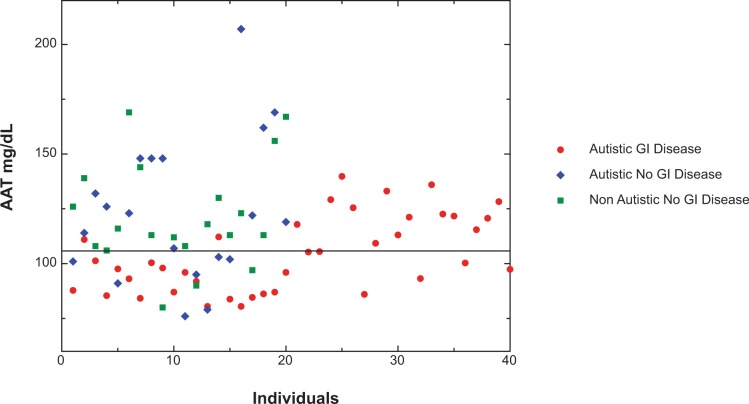
Scatter diagram of Alpha-1 antitrypsin (AAT) concentrations in the serum of 40 autistic children with severe GI disease and 41 controls (21 age (mean 68 months), gender (80% male) and diagnosis (61% regressive onset) matched autistic children with no GI disease and 20 age (mean 71 months) and gender (75% male) matched children without autism or GI disease). Normal levels of AAT are 85–210 mg/dL serum. The data points represent the mean of at least 2 separate assays. The standard deviation (not shown) of each mean was less than +/− 0.01 OD.

**Figure 3:**
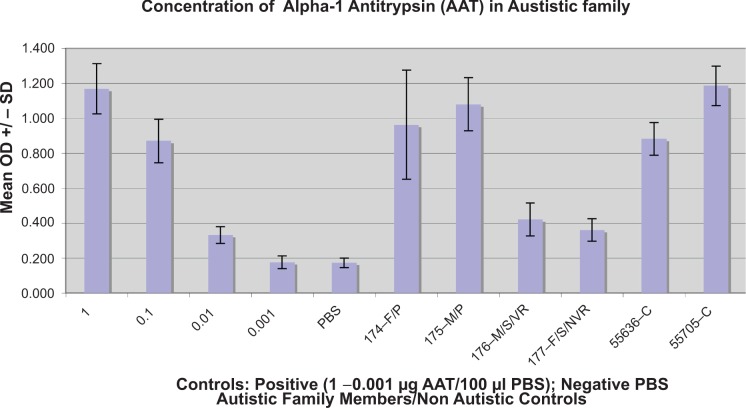
Results of a typical ELISA establishing Alpha-1 Antitrypsin (AAT) serum concentration of autistic and control family members, using purified AAT (Sigma) and monoclonal anti-AAT IgG (Biomedia). Positive control, purified AAT (1 μg–0.001 μg) on right. Negative control (PBS), far right. Non Autistic controls, parents with no family history of autism. Each Mean OD +/− SD was established from 4 samples (wells).

**Figure 4:**
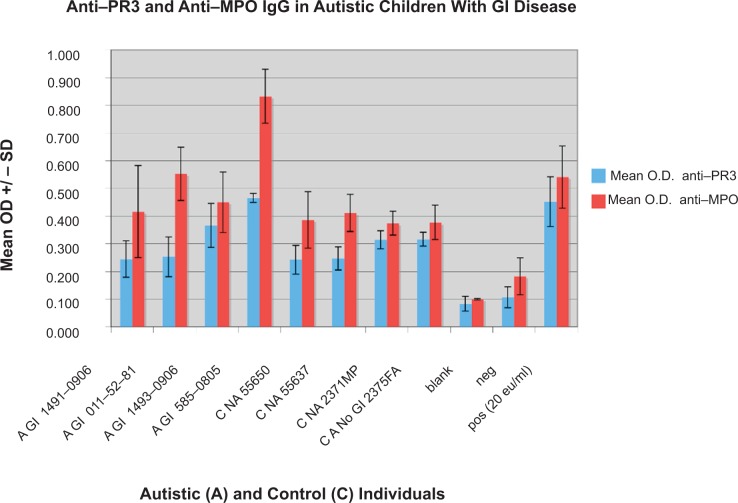
Results of a typical ELISA measuring the presence of anti-MPO IgG in an autistic (A) child with GI disease (GI), A GI 011-52-81, and both anti-MPO and anti-PR3 IgG in an autistic (A) child with GI disease (GI), A GI 585-0805. None of the controls (C NA—non autistic; C A—autistic without GI disease) have anti PR3 or anti-MPO antibodies. Each Mean OD +/− SD was established from 4 samples (wells).

Using indirect ELISAs, described above, we measured serum levels of AAT and PR3. Twenty four of the 40 autistic children with chronic digestive disease also had low serum levels of AAT (<= 105 mg/dL), compared to ten of 41 controls tested for serum AAT (p < 0.05) (Fig. 2). Seven of these 10 were autistic children with no GI disease. Figure 3 shows the results of a typical assay measuring serum AAT levels in autistic children with GI disease.

**Figure 5:**
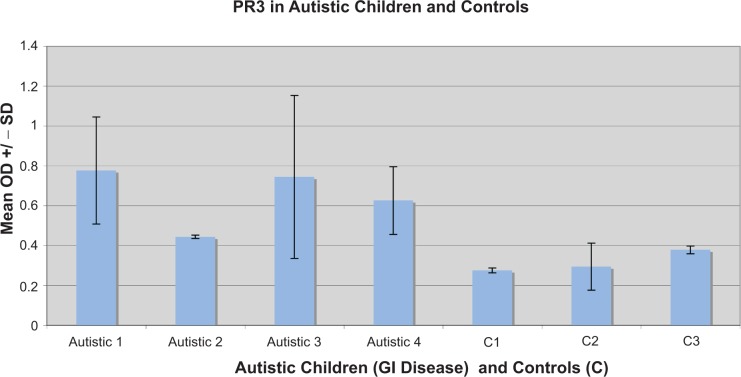
Results of a typical ELISA measuring the presence of PR3 in autistic children with GI disease and controls (C). Control 1 and 2 are autistic children with no GI disease. Control 3 is a child with no autism or GI disease. Each Mean OD +/− SD was established from 4 samples (wells).

Twelve of the 40 autistic children with GI disease had high levels of PR3 (>= mean of 18 normal controls), Eight of these 12 had low serum AAT (Table 1) (p < 0.05). All 6 with anti-PR3 ANCA had high levels of PR3 (p < 0.01). Six of 12 with high PR3 levels also scored high with respect to GI disease severity (p < 0.05) (Table 1). Figure 5 shows the results of a typical assay measuring serum PR3 levels in autistic children with GI disease.

There was also a significant relationship between individuals with low AAT and severity of intestinal disease, as 11 out of 15 with severe intestinal disease also had low serum AAT (p < 0.05), 5 of 6 with severe LNH also had low AAT (p < 0.05), and 5 out of 5 with severe erythema had low serum AAT (p < 0.05). All 3 individuals with the most severe levels of GI disease had high serum PR3 levels (Table 1).

## Discussion

Our results show that autistic children with severe GI disease have low serum levels of alpha-1 antitrypsin, and the data suggests that these low concentrations are associated with the presence of ANCA and high levels of PR3.

AAT is a naturally occurring inhibitor of proteinase 3 (PR3), one of the target antigens of anti-neutrophil cytoplasmic antibodies (ANCA). Individuals with abnormal AAT phenotypes have a significantly higher chance of having ANCA (particularly PR3),^30^,^31^,^34^ and low serum AAT levels are associated with anti-PR3 in patients with severe vasculitis and pulmonary hemorrhage.^33^ Abnormal AAT phenotypes have also been associated with severity of ANCA associated disease.^35^

There has also been a report of an association between abnormal AAT phenotypes in patients with ANCA and microscopic polyarteritis.^34^

Why anti-PR3 antibodies occur more often in patients with abnormal AAT phenotypes is not clear. AAT is usually complexed to PR3 in the circulation and in secretions.^32^ It binds to the catalytic site of PR3 and can inhibit binding of this enzyme to its substrate and thus interfere with its enzymatic activity.^35^ Individuals with deficient or abnormal AAT may have a PR3 molecule whose catalytic site is exposed. This might result in a new molecular entity which is recognized by the subjects immune system as foreign, resulting in the production of antibodies (autoantibodies).

Our results suggest that autistic children with severe GI disease, characterized by intestinal inflammation, have low serum levels of AAT, which in turn is associated with higher than normal levels of PR3 and the presence of ANCA.

Since PR3 is a major serine protease released from neutrophils and monocytes at local sites of inflammation and tissue injury, and a major role of alpha-1 antitrypsin(AAT) is to inhibit the destructive capabilities of PR3, and that AAT acts as a serine protease which degrades PR3 and therefore would result in lower serum PR3, we suggest that in these autistic children, a deficiency of AAT results in an increase in blood levels of free and active PR3 which, in turn, generates further tissue injury and an immune response resulting in the formation of ANCA.

ANCA, in turn, may compound PR3-related vascular injury by generating a cascade, which includes activation of tumor necrosis factor (TNF)-primed neutrophils, acceleration of apoptosis of TNF-primed neutrophils, and secondary necrosis of TNF-primed neutrophils. These may contribute to the intestinal inflammation observed in many of the autistic children with GI disease.

## Figures and Tables

**Table 1:** Comparison of ANCA (anti-PR3), AAT serum concentration, PR3 serum levels and severity of GI Disease, in autistic children with severe GI disease. Those in Bold or Red represent high levels of AAT, ANCA and/or PR3. Patients were scored according to mild (1 point), moderate (2 points) and marked (3 points) disease in each area (upper and lower GI) and for scope (macroscopic) and histology of each area. Therefore the maximum score for GI disease would be 12 (3 points each for upper scope, upper histology, lower scope and lower histology). A point system was also developed for severity of lymphoid nodular hyperplasia (LNH). Patients were scored according to mild (1 point), moderate (2 points) and marked (3 points) LNH in each area (upper and lower GI) for a maximum of 6 points. And finally, a point system was also developed for severity of erythema. Patients were scored according to mild (1 point), moderate (2 points) and marked (3 points) erythema in each area (upper and lower GI) for a maximum of 6 points.

Diagnosis	Mean O.D. anti-PR3**	Mean O.D. AAT	AAT mg/dl***	Mean O.D. PR3****	LNH	Eryth	Total GI
**Autistic individuals with GI disease**							
RA	0.231	**0.351**	**87.8**	0.418	2	0	5
A	0.140	0.444	111.0	0.389	3	0	6
A	0.214	**0.405**	**101.3**	**0.277**	3	**6**	**9**
R-PDD	0.245	**0.342**	**85.4**	0.385	1	0	3
RA	0.253	**0.390**	**97.6**	0.411	1	0	3
RA	0.366	**0.372**	**93.1**	**0.332**	2	2	6
RA	**0.466**	**0.337**	**84.2**	0.444	**4**	**3**	**8**
RA	0.134	**0.402**	**100.4**	0.453	2	0	**8**
R-UD	0.251	**0.392**	**98.0**	0.407	**4**	2	**8**
RA	0.147	**0.348**	**87.0**	**0.331**	3	1	**7**
A	0.129	**0.384**	**96.0**	0.433	3	1	6
RA	0.121	**0.368**	**91.9**	0.385	1	0	**7**
PDD/NOS	0.181	**0.322**	**80.5**	**0.267**	2	2	6
A	0.189	0.449	112.2	0.363	1	2	6
A	**0.401**	**0.335**	**83.8**	0.458	3	**5**	10
RA	0.196	**0.322**	**80.5**	0.448	0	2	5
A	0.179	**0.338**	**84.6**	0.409	2	1	5
RA	0.175	**0.345**	**86.2**	0.429	3	1	NA
R-PDD	**0.443**	**0.348**	**87.0**	0.512	**5**	0	NA
A	**0.576**	**0.384**	**96.0**	0.777	**4**	**4**	**11**
R-PDD	0.278	0.472	117.9	0.451	3	1	**8**
RA	0.199	**0.421**	**105.3**	0.431	3	2	5
RA	**0.544**	**0.422**	**105.5**	0.449	3	0	**7**
A	0.218	1.717	429.2	0.400	3	2	**8**
RA	0.140	0.559	139.8	0.386	2	0	4
RA	0.259	0.502	125.5	0.475	3	0	4
R-ASP	0.099	**0.344**	**86.0**	0.432	2	1	6
A	0.174	0.437	109.3	0.456	3	**4**	6
PDD	0.197	0.532	133.1	**0.325**	2	1	4
R-PDD/NOS	0.274	0.453	113.1	0.745	2	1	4
RA	0.235	0.485	121.2	0.412	3	0	6
A	0.233	**0.373**	**93.2**	0.412	2	0	4
RA	**0.482**	0.544	136.0	0.442	3	2	6
A	0.136	0.491	122.6	**0.276**	0	0	NA
RA	0.227	0.487	121.7	**0.295**	3	0	5
RA	0.221	**0.401**	**100.3**	0.413	2	2	**7**
RA	0.141	0.462	115.5	0.411	2	0	3
A	0.173	0.483	120.7	0.417		0	**7**
RA	0.166	0.513	128.3	0.403	3	2	**7**
RA	0.289	**0.390**	**97.4**	0.626	**6**	0	**10**

* >0.4 O.D. = positive Anti-PR3 antibodies >= 20 Eu/ml.

** Alpha-1 Antitrypsin (AAT) mg/dL; bold =< 110 mg/dL (85–210 mg/dL is normal range); Mean of 18 controls = 135 mg/dL +/− 22 mg/dL.

*** Mean O.D. of 18 controls = 0.442 =/− 0.053.

**Abbreviations:** D, Diagnosis R, regression; A, autistic; UD, undetermined.

## Footnotes

* The Autism Genetic Resource Exchange (AGRE) is the first collaborative gene bank for the study of autism spectrum disorders and one of the world’s largest shared resources for the study of autism and related disorders, with a collection of over 900 well-characterized multiplex and simplex families made available to the greater scientific community. Founded by Cure Autism Now (CAN) in 1997, AGRE is currently funded by the National Institute of Mental Health (NIMH) and Autism Speaks (AS), which merged with CAN in 2006.
